# Why Men Matter: Mating Patterns Drive Evolution of Human Lifespan

**DOI:** 10.1371/journal.pone.0000785

**Published:** 2007-08-29

**Authors:** Shripad D. Tuljapurkar, Cedric O. Puleston, Michael D. Gurven

**Affiliations:** 1 Department of Biological Sciences, Stanford University, Stanford, California, United States of America; 2 Department of Anthropology, University of California at Santa Barbara, Santa Barbara, California, United States of America; University of Wisconsin, United States of America

## Abstract

Evolutionary theory predicts that senescence, a decline in survival rates with age, is the consequence of stronger selection on alleles that affect fertility or mortality earlier rather than later in life. Hamilton quantified this argument by showing that a rare mutation reducing survival is opposed by a selective force that declines with age over reproductive life. He used a female-only demographic model, predicting that female menopause at age ca. 50 yrs should be followed by a sharp increase in mortality, a “wall of death.” Human lives obviously do not display such a wall. Explanations of the evolution of lifespan beyond the age of female menopause have proven difficult to describe as explicit genetic models. Here we argue that the inclusion of males and mating patterns extends Hamilton's theory and predicts the pattern of human senescence. We analyze a general two-sex model to show that selection favors survival for as long as men reproduce. Male fertility can only result from matings with fertile females, and we present a range of data showing that males much older than 50 yrs have substantial realized fertility through matings with younger females, a pattern that was likely typical among early humans. Thus old-age male fertility provides a selective force against autosomal deleterious mutations at ages far past female menopause with no sharp upper age limit, eliminating the wall of death. Our findings illustrate the evolutionary importance of males and mating preferences, and show that one-sex demographic models are insufficient to describe the forces that shape human senescence.

## Introduction

**Figure 1:**
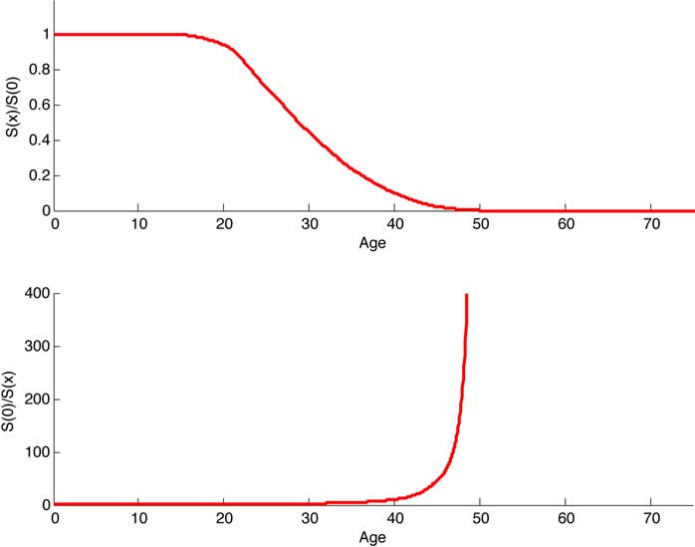
The wall of death. This figure shows the force of selection by age, *S*(*x*), as a fraction of the force of selection at birth, *S*(0), as described by Hamilton using female-only demography. (a) Hamilton's one-sex force of selection at age *x* (shown relative to its value at birth) falls to zero with the decline of remaining survival-weighted female reproduction. b is the inverse of the top panel. In mutation-selection balance, the frequency of deleterious mutant alleles is expected to be proportional to 1/*S*, where *S* is the force of selection for a dominant or semi-dominant allele. The inverse of the force of selection is an indicator of age-specific mortality. The rapid increase in mortality at female menopause is the ‘wall of death.’

Evolutionary theory [1], [2] predicts that human survival rate declines at old ages because selection against mutations that reduce survival weakens with age. Hamilton [1] used one-sex (female) demography [3] to show that selection against a rare mutation at an autosomal locus that reduces survival at any age is proportional to a female's expected survival-weighted reproduction past that age. Thus selection to maintain survival should decline with age, favoring pleiotropic alleles that have positive effects at young ages and negative effects at older ages, and allowing alleles that are simply deleterious at old ages to reach a high frequency. Because selection to maintain survival should fall to zero after female menopause by age 55 yrs [4], accumulation of mutations that reduce old-age survival rates should lead to a sharp rise in mortality at female menopause (Fig. 1), aptly called a “wall of death.” But 31% of people live past age 55 yrs in human hunter gatherer populations (life expectancy 33.5 yrs) [5]. Life expectancy in today's industrialized countries is 75–85 yrs [6], and mortality increases gradually, not suddenly, with age after female menopause [4].

Ecological explanations for lifespan beyond the age of female menopause have focused on the value of transfers from the old to the young as measured by gains in fitness. Hawkes [7] and colleagues made the qualitative argument that grandmothers advantage their daughters and granddaughters through care of grandchildren. Lee [8] makes a quantitative analysis of the fitness benefit of transfers between age groups. Kaplan and Robson [9] argued that the presence of older people maximizes fitness as measured by economic efficiency, and Shanley and Kirkwood [10] argued that older females enhance the survival of young. Support for these explanations of observed patterns of senescence is in the form of correlations [7], [9], or simulations [8]. But these studies are not framed as models that track the gene frequency of rare survival-altering mutations, and thus do not yet extend Hamilton's framework. In contrast, Rogers [11], tracks gene frequency change in asking whether a tradeoff between a female's future reproduction and the enhanced survival of her offspring could explain the evolution of menopause. But his results are inconclusive and we conclude that such tradeoffs alone are not likely to explain female patterns of senescence.

In the context of Hamilton's theory, Charlesworth [12], [13] and Marlowe [14] suggested that senescence may be keyed to the fact that human males can reproduce at high ages. But reproductive potential does not imply reproductive fitness: the latter must derive indirectly from the reproductive fitness of females. While there have been major steps forward in our understanding of the evolution of senescence, Medawar's “unsolved problem of biology” [15] remains so.

Here we show that a great part of the problem is resolved by adding realistic patterns of mating and the resulting male fertility to Hamilton's approach: This can only be done using a two-sex model. We first present data showing that observed male fertility in many human populations is nonzero between the ages of 55 and 70. This pattern and its implications were first discussed by Marlowe [14] using data on Tanzanian Hadza hunter-gatherers. But we must still prove that older males contribute to selection at ages greater than female menopause. A male-only analysis [12] cannot answer this question, and does not change Hamilton's female-only results. Unlike the reproduction of clonal organisms, human mating patterns depend on the age and sex structure of populations and on culturally defined rules of pairing. Models of human evolution without these elements can yield mistaken conclusions about evolutionary processes [16].

Here we analyze the change in frequency of a rare autosomal mutation in a two-sex demographic model, showing that selection against autosomal mutations that reduce survival after the age of female menopause is proportional to remaining male survival-weighted reproduction. We derive expressions for the two-sex force of selection as a function of population structure and mating pattern. Our analysis shows that old-age male fertility allows evolution to breach Hamilton's wall of death and predicts a gradual rise in mortality after the age of female menopause without relying on “grandmother” effects or economic optimality.

## Results

**Figure 2:**
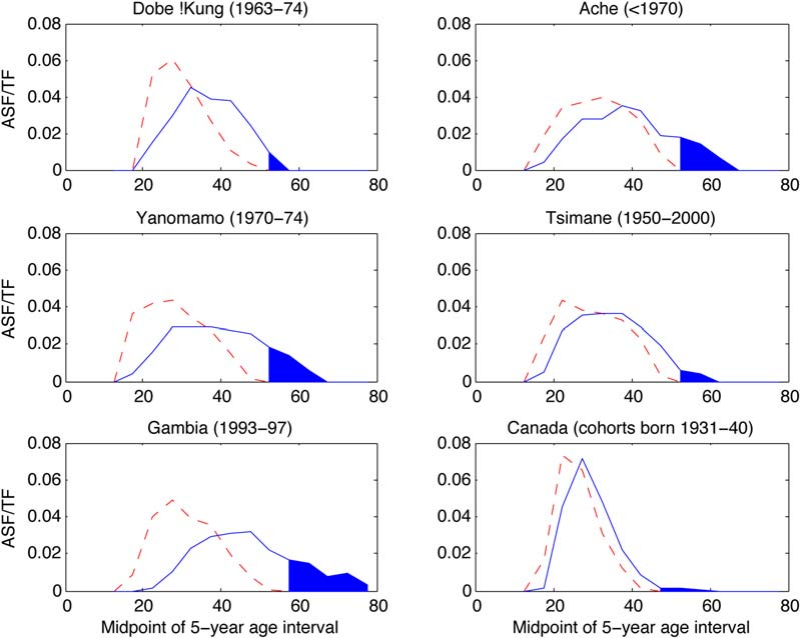
Observed distributions of female and male fertility. Fertility distributions (in age-specific fertility rates as a fraction of total fertility rate) for women (dashed red) and men (solid blue) for (a) the hunter-gatherer Dobe !Kung of Botswana, (b) the forest-living Ache, (c) the Amazonian forager-horticulturalist Yanomamo, (d) Bolivian forager-horticulturalists the Tsimane, (e) agricultural Gambian villagers, (f) modern Canada. The blue shaded area represents realized male fertility after the age of last female reproduction.

**Figure 3:**
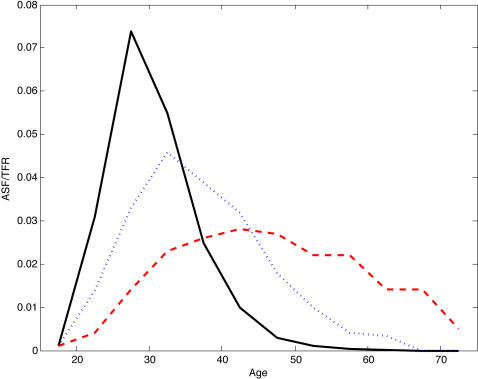
Three distinct male fertility distributions. Male fertility in1980 France (black), Pakistan 1984 (blue dots) and Cameroon 1964 (red dashes). Redrawn from Paget and Timaeus [24] Cameroon's distribution is common of high-fertility polygynous societies. The Y-axis shows age-specific fertility rates as a fraction of the total fertility rate.

The male fertility rate at any age is the ratio of all births to pairs involving a male of that age to the number of males of that age. Fig. 2 shows scaled age-specific fertility for (a) the Dobe !Kung, a hunter-gatherer group in the Kalahari [17], (b) the Ache, a hunter-gatherer society in Paraguay that during the study period was one of the most isolated populations in the world (data for the forest-living Ache [18]), (c) the forager-horticulturalist Yanomamo of Brazil and Venezuela [19], (d) the Tsimane, an indigenous forager-farming group in Bolivia [20], (e) a group of agricultural villages in the Gambia [21], and (f) modern Canada [22], for comparison. Male fertility is nonzero till ages 55 yrs in Canada and the !Kung, 65 yrs in the Ache, 70 yrs in the Yanomamo, 60 yrs in the Tsimane, and 75 yrs in the Gambia. The populations in Fig. 2 a–d likely represent early human demographic conditions and mating patterns [23]. Late male fertility is also found in national populations: Paget and Timaeus [24] used 1960–1999 data to derive a standard male fertility which is nonzero from ages 55 to 80 yrs. Fig. 3 (redrawn from Paget and Timaeus [24]) shows nonzero fertility to age 75 in the Cameroon in 1964, similar to the Gambia, and to age 65 in Pakistan in 1984. Kuhnert and Nieschlag [25] show that in Germany (in 2001) and Japan (in 2002) males at age 65 had realized fertility equal to that of females aged 45.

Late male fertility derives from diverse cultural patterns of mating, including age gaps at marriage, serial monogamy and polygyny. Universally, older males marry younger females (by 5–15 yrs in less-developed, traditional societies [26]). The mating age gap is most pronounced in societies that favor polygyny [27], or a gerontocracy [14], in which old men monopolize access to reproductive females. Polygyny is common in Cameroon [28] and the Gambia [21], and occasionally observed in the Ache [18], !Kung [17], Tsimane [20], and Pakistan[29]. Late-age male fertility characterizes several other African countries [30]. Additionally, molecular evidence [31] and studies of human sexual dimorphism and testes size [32] suggest that humans were polygynous through much of our evolutionary history. Late age male fertility also results from serial monogamy, because men are more likely to remarry than women. High-fertility populations like the Ache, sub-Saharan African populations and Pakistan disperse male fertility over a wider (and later) span of ages [24]. For these reasons, we argue that realized male fertility was substantial at ages well past female menopause for much of human history and the result is reflected in the mortality patterns of modern populations.

What effect does a stable (over time) mating pattern, in which older males have fertility past the age of female menopause, have on the fate of late age survival-reducing mutations? We use two-sex demography [33], [34] and, following Hamilton [1], examine the fate of a rare mutation that affects mortality past the age *K* ≈ 55 yrs of female menopause. The mutation arises at an autosomal locus that affects survival rate in both sexes at a age *J*>*K*, past the age of last female reproduction. Initially a population contains only individuals homozygous for an allele A at this locus; a rare mutant allele B changes male survival from *p_J_^M^* for the AA genotype to *p_J_^M^* (1+*δ*) for the AB genotype (with *δ* very small). The initial rate of increase of allele B is determined by the strength of selection *S = r_AB_-r_AA_* where *r_AB_, r_AA_*, respectively are the stable growth rates of populations made up entirely of individuals with the AB, AA phenotypes. With female-only demography, a post-menopausal mutation has no effect on population growth rate and *S = 0*.

Two-sex population dynamics depend on the age distributions of males and females. Denote female and male survivorships from birth to age *i* by, respectively, *l^F^*(*i*), *l^M^*(*i*). We assume that the two-sex model satisfies standard demographic assumptions [33] (see Methods). A demographically locally stable equilibrium population [33] of AA genotypes grows at rate *λ = *exp(*r_AA_*) per unit of time, the stable female age structure is {*λ_AA_^i^* ^+1^ *l_i_^F^*}, and the stable male age structure is *σ*{*λ_AA_^i^* ^+1^ *l_i_^M^*}. Here *σ* is the male-to-female sex ratio at birth. In a two-sex model at equilibrium age-specific reproduction is described by marginal fertilities (at age *n*, *G_n_^F^* for females, *G_n_^M^* for males). Note that marginal fertilities are not equal to the fertilities in any one-sex model, are functions of the population's age-sex composition and mating rules, and that male and female marginal fertilities are usually very different (see Appendix S1). We assume that male marginal fertility is positive at any age where realized male fertility is nonzero (this assumption holds for all standard models of mating pair formation [34]).

The stable growth rate of a population with the AB phenotype is *λ*_1_ *= *exp(*r_AB_*)* = *exp(*r_AA_+S*); since the mutant allele B has small effect we know that *S* is small. Computation (see Methods) shows that 
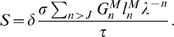
(1)Here *σ* is the sex ratio at birth, and *τ* is the generation time 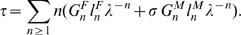



In a female-only model, *S = 0* regardless of whether *δ*>0 or <0. In a male-only model, *S* is independent of mating patterns and female population composition. Biologically, when *δ*<0, *S* is the loss of fertility that results because there are fewer males older than *J* years, so we lose the offspring of matings between these males and all females. Hence the strength of selection *S* is proportional to the expected reproduction by males at ages older than *J*, which equals Σ*_n>J_G_n_^M^l_n_^M^λ^−n^*. So long as those males have a nonzero fertility, their reproduction after age *J* will be positive. We conclude that deleterious mutations acting after the age of female menopause (*δ<0*) are selected against because *S<0*, solely as a result of the matings between older males and younger females.

**Figure 4:**
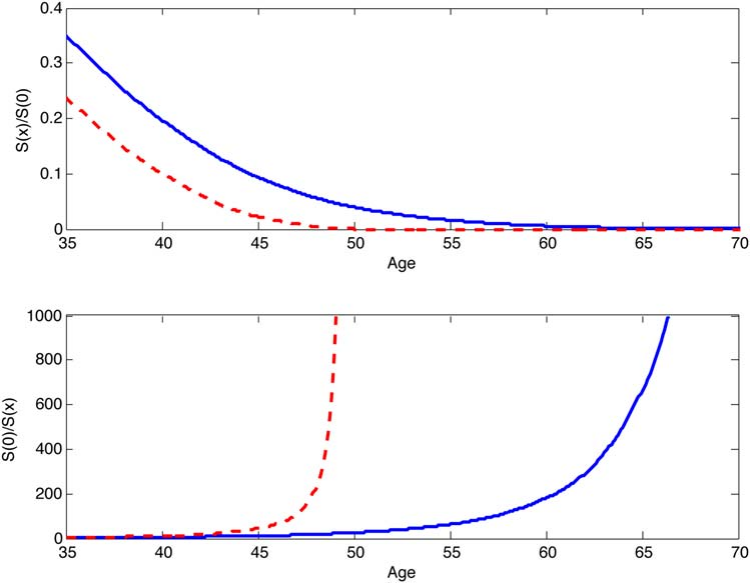
Two-sex model eliminates wall of death. In a the dashed red line shows the relative force of selection (as in Fig. 1) on a forager population with a life expectancy of 33.5 years using female-only demography. The solid blue line shows the two-sex relative force of selection and reveals an active defense against deleterious mutations into the 65–70 yr age interval. b shows the inverse of the force of selection, which describes the pattern of mortality. The wall of death is replaced by a gradual rise in mortality until very late ages.

Fig. 4 shows the age pattern of selection predicted by equation (1). In contrast to the female-only prediction of zero selection after female menopause, the two-sex model predicts a selection pressure that persists to a much later age. The strength of selection in Fig. 4 will vary with the realized fertility of older males, however, the general pattern will be expected to persist given any mating age gap favoring older males.

## Discussion

Our analysis (see Appendix S1) shows that the age-specific force of selection is a weighted average of the remaining survival-weighted reproduction in each sex. Equation (1) tells us that after the age of last female reproduction the Hamiltonian force of selection is derived entirely from the pattern of male reproduction with younger females. Our analysis of mating patterns shows that productive mating between men older than the age of female menopause and younger women was likely a feature of early human life. Even when life was much shorter than today there was a reasonable supply of older males: among hunter gatherers with an expected lifespan of 33.5 yrs, the ratio of 70 yr olds to 30 yr olds was about 0.32 and to 40 yr olds was about 0.37 [5]. Therefore natural selection should have acted against survival-reducing mutations and delayed the onset of rapid senescence for as much as two decades past female menopause.

At still older ages, we predict that mortality rates should continue to rise gradually, rather than abruptly like a wall of death, for four reasons. First, male fertility does not fall abruptly at a specific age; robust males (such as chiefs or high-status males [23], most notably in Australian aboriginal societies [14]) could have maintained fertility at relatively high ages. Second, older male fertility would have been higher at times when younger males faced high mortality from, e.g., warfare or hunting effort in lean times. Third, Charlesworth [12] showed that if some genes are beneficial at both reproductive and post-reproductive ages, then mutations that damage those genes should be selected against and late-life mortality should be restrained as a result. Finally, the fundamental two-sex force of selection we describe here may well be enhanced by intergenerational transfers [7], [8], [9].

The inclusion of male reproduction has two important effects on predictions of senescence, the first a consequence of the mean age of male reproduction and the second a consequence of the shape of the distribution. If the pattern of male reproduction were identical to that of females but shifted to the right, the wall of death would still exist, but with a step-like shape between the years of last female and last male reproduction. However, the typical male and female fertility distributions are not identical in shape. The long tail of the male fertility distribution forestalls the rapid increase in mortality that Hamilton predicted and slows the rise towards infinity.

## Methods

We use Pollak's [33] and Schoen's [34] expositions of two-sex demography. Female numbers by age *i* are **F** = {*F_i_*}, male numbers are **M** = {*M_i_*} at ages *i*. Matings between a female aged *i* and a male aged *j* produce on average *B_ij_* female births in one time interval. The functions *B_ij_*(**F**,**M**) satisfy standard demographic assumptions [33]. All else fixed, births from *i,j* matings increase with the number of potential mates *F_i_* and *M_j_*. We assume unions are formed in each period; persistent unions would complicate the analysis but not change the conclusion [33]. Marginal fertilities *G_n_^F^* for females are 
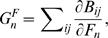
(2)evaluated at the stable equilibrium (see Appendix S1); *G_n_^M^* for males is defined analogously. Male marginal fertility at age *n* is nonzero in general. The characteristic equation for stable growth is 
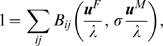
where **u** *^F^* and **u** *^M^* are the stable female and male age structures, and *σ* is the male-to-female sex ratio at birth.

Euler's theorem on homogeneous functions allows us to rewrite this as 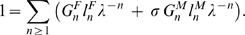
(3)The result (3) is obtained by inserting the changes in survivorship and growth rate of an AB phenotype into the characteristic equation, and then using a standard Taylor expansion.

The example in Fig. 4 is constructed by using a form of Schoen's harmonic mean model [34] in which 
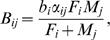
the *b_i_* are fertility levels for females aged *i*, and the *α_ij_* are mating preference weights. We constructed these weights from age gaps of marriage among the Dobe !Kung [17], and used empirical hunter-gatherer life tables assembled by Gurven and Kaplan [5] with life expectancy 33.5 yrs. Female fertility distribution is chosen to represent hunter-gatherers and derived from the Ache and !Kung. Stationary populations for one-sex and two-sex models are computed in the usual way.

## Supporting Information

Appendix S1 (0.45 MB DOC)
