# Supplementary material for: Why Men Matter: Mating Patterns Drive Evolution of Human Lifespan
Source: PLoS One. 2007 Aug 29;2(8):e785. doi: 10.1371/journal.pone.0000785 (PMC1949148; doi:10.1371/journal.pone.0000785)
Supplement: Appendix S1 — (0.45 MB DOC) [file pone.0000785.s001.doc]

# Appendix S1

## Two-Sex Demography

We follow Pollak [33] and use a model of two sex demography in which females and males are enumerated in discrete age classes and population dynamics are followed in discrete time . We describe reproductively successful matings in each period between females aged and males aged by the fertility (number of female offspring) of such matings given by a birth function . This function is assumed to satisfy the standard properties of such a pair-reproduction function [33]. The ones that matter here are that is homogeneous of degree 1 in its arguments and is nondecreasing in its arguments. Two further conditions are specified later on. An example of such a function is Schoen’s [34] harmonic mean function, which will also be discussed later.

The contribution of males to the realized fertility of a female age is determined by population composition and preferences. In the general model with a given population structure, the fertility of all females aged is and the fertility of males aged is . For simplicity we assume that all reproductive unions last for one time period; persistent unions can be tracked using the methods in Pollak [35] but do not alter our central argument.

Given population numbers in year , female births in year are given by . Male births occur in fixed proportion to female births, assuming a fixed sex ratio at birth as is characteristic of humans. Sex-specific survival rates by age are with corresponding survivorships . These survivorships determine population composition in terms of past births.

We assume that the birth function generates a locally stable equilibrium population growing at exponential rate per unit of time. We use the term stability in its demographic sense, meaning that the relative abundances by age remain constant even as the population grows. At this equilibrium, female births at and satisfy . The equilibrium age structures of females and males at time can be written as with and with . Substituting into the expression for female births gives . The homogeneity of the birth function allows us to divide all terms by

(A−1)

It is important to note that the only ages that enter into equation (A-1) are female ages up to menopause, and all male ages that are involved in reproductively successful matings.

## Selection in Two-Sex Demography

Consider two alleles A, B at a single autosomal gene locus that affect some aspect of a two-sex age-structured life history, for example, the survival rate of one or both sexes. To analyze selection in this situation we need to work with genotype numbers or frequencies for the AA, AB and BB genotypes, rather than gene frequencies, as is the case for most models of fertility selection [36]. We are interested only in alleles that affect individual genotypes via survival and not the potentially more complex effects of genotypic change on the properties of genotype pairs via the birth function. Suppose now that a rare allele B is introduced into a population that is largely homozygous for allele A. Standard arguments [3] show that in this case the rare allele will invade if the growth rate of the heterozygous genotype AB exceeds that of the common genotype AA. Thus we need only compare the equilibrium growth rates of populations with the AA and AB phenotypes, and the difference in growth rates determines whether B invades.

## Change in Growth Rate when Survival Changes

Let be an upper bound on the age at female menopause; there is only small variation in the age at female menopause so a bound at age 55 will suffice. Let be the highest age of males who are involved in reproduction; consistent with the data we have . We start with a population homozygous at an autosomal locus which affects survival rate in both sexes at some age with and . This homozygous population has a stable growth rate determined by equation (A-1). Assume that a mutation in heterozygous form changes male survival rate at age from to with a small . Male survivorship will be unaffected at ages less than and at ages will change to . Female survivorship at all ages up to are unchanged by this mutation. The growth rate of a population of heterozygotes will be with small. To compute we apply (A-1) to this heterozygous population. The stable age structure in this population differs from the homozygous population because of the change in growth rate and the change in survivorship; call the new structures . Since will be of order , we track changes to order . For females at ages through ,

For males at ages through ,

For males at ages and higher,

For simplicity let stand for the change in in sex S.

Finally we require partial derivatives of the birth function evaluated at the stable equilibrium of the original homozygous population. We write

(A−2)

The partial derivatives in (A-2) are all greater than or equal to zero. Inserting these expressions into equation (A-1) yields the result

Replacing the shorthand s, multiplying through by and rearranging gives the result

(A−3)

For the left side of this equation, recall that is homogeneous of degree 1, so we can use equation (A-1) and Euler’s homogeneous function theorem to write

This implies that the bracketed expression multiplying on the left of (A-3) is bounded below by and above by where is the highest age of males or females who are involved in reproduction. These bounds show that

On the right side of equation (A-3), the partial derivative will be positive if an increase in the number of males increases the births that result from matings between males aged and females of some reproductive age . Because the birth functions are nondecreasing in their arguments, the right hand side of (A-3) is greater than or equal to zero. When mating preferences produce a significant number of reproductive matings between males of ages greater than the age at female menopause and fertile women, we will have for some ages . Our data show that such mating preferences will hold in many human populations. Then the curly bracketed terms on both sides of equation (A-3) will be positive and the change in growth rate will be positive if and vice versa. We have thus demonstrated that there will be selection against a mutation that reduces male mortality at ages past the age of female menopause.

In fact the bracketed expression multiplying on the left of (A-3) is a generation time similar to the mean length of generation in one-sex demography. The partial derivatives in (A-3) are marginal fertilities: thus is the effect on the total number of births of an increase in the number of females of age , and is the effect on the total number of births of an increase in the number of males of age . Thus we can define a generation time as an average age weighted by the product of marginal fertilities and stable age proportions,

(A−4)

which we call the two-sex generation length. In equation (A-3), the square bracketed term on the left is just . The definition of the marginal fertilities also shows that the term in curly brackets on the right of (A-3) can be rewritten as

(A−5)

where is the change in the number of births resulting from the change in the number of males above age that is produced by the mutation. Thus the strength of selection reduces, sensibly, to

(A−6)

## Harmonic mean model

Schoen [34] described the harmonic mean model of mating, a version of which can be written

(A-7)

with . In this function is the culturally determined propensity of a female age to have a female baby with a male age , and is the potential fecundity of a female aged . Male potential fecundity is assumed constant from puberty until death. Equation (A-7) yields an explicit version of equation (A-3). In the harmonic mean model depends only on females age and males age , so that

(A-8)

and

(A-9)

where we introduce the symbol

(A-10)

and we are using the Kronecker delta ( if and else is zero). Inserting these into the expression that multiplies on the left of (A-3) yields

and

These expressions identify the marginal fertilities explicitly as

(A-11)

and

(A-12)

These marginal fertilities can now be inserted into equation (A-4) and then into equation (A-6) to obtain in terms of .
